# Frequency of nasal carriage of *Staphylococcus Aureus* among health care workers at a Tertiary Care Hospital

**DOI:** 10.12669/pjms.345.14588

**Published:** 2018

**Authors:** Muhammad Kashif Salman, Muhammad Sohail Ashraf, Sumaira Iftikhar, Mirza Ahmad Raza Baig

**Affiliations:** 1 Muhammad Kashif Salman, MBBS, FCPS (Pediatrics Resident), Nishtar Medical University/Hospital Multan, Pakistan; 2 Muhammad Sohail Ashraf, MBBS, Nishtar Medical University/Hospital Multan, Pakistan; 3 Sumaira Iftikhar, MBBS, FCPS (Gynecology), Nishtar Medical University/Hospital Multan, Pakistan; 4 Mirza Ahmad Raza Baig, B.Sc (Hons), Clinical Perfusion Specialist, Cardiac Center at Hail Region, Hail, Saudi Arabia.

**Keywords:** Health Care Workers, *Methicillin resistance staphylococcus aureus*, Nasal carriage

## Abstract

**Objective:**

To determine the frequency of *Methicillin resistance staphylococcus aureus* (MRSA) and identification of drug susceptibility for MRSA isolates among health care workers (HCWs) of a tertiary care hospital of South Punjab Pakistan.

**Methods:**

We included 225 HCWs including laboratory staff, doctors, nurses and paramedical staff in this cross-sectional study. The study was conducted in Nishtar medical university/Hospital Multan. The study duration was July-2016 to April-2017. HCWs having no signs of infections and any other systemic disease were included in this study. We used sterile nasal swab sticks for sample collection for determination of *S. aureus prevalence*. All these samples were processed in the laboratory for MRSA, *methicillin sensitive S. aureus* (MSSA) and for antimicrobial sensitivity of *S. aureus*. Chi-square test was used for comparison of frequency of MRSA and MSSA between different HCWs by assuming p-Value ≤0.05 as significant difference.

**Results:**

There were 65.3% (147) female participants and only 34.7% (78) male participants. *S. aureus* was diagnosed in the nasal flora of 24% (54) participants, out of which 9.3% (21) were MRSA positive and remaining 14.7% (33) were MSSA positive. There was no significant difference in frequency of MRSA and MSSA among different HCWs (p-value 0.79). Amikacin and vancomycin were 100% sensitive for MRSA and MSSA. Clindamycin and ciprofloxacin was 80.9% (17) and 71.4% (15) sensitive for MRSA and 100% and 84.8% (28) for MSSA respectively. While oxacillin and Cefoxitin were 100% (21) resistant for MRSA and sensitive for MSSA.

**Conclusion:**

Prevalence of MRSA and MSSA is high among HCWs in Pakistan. Amikacin, vancomycin and clindamycin have high sensitivity for MRSA and can be used for empirical treatment of MRSA in suspected patients.

## INTRODUCTION

*Staphylococcus aureus* is one of the leading cause of infectious diseases including many skin and soft tissue diseases, respiratory tract infections, meningitis, endocarditis, urinary tract and wound infections.^1^,^2^ *Methicillin resistant Staphylococcus Aureus* (MRSA) is a leading cause of hospital acquired as well as community acquired infections not only in humans but also other mammalians.^3^,^4^ It is a major cause of wound contaminations and other invasive infections in hospitalized patients thereby increasing morbidity and mortality in these patients.^5^,^6^ MRSA are strains of *S. aureus* that are resistant to methicillin and other beta-lactam antibiotics.

Nasal flora of MRSA have been proven to play an important role in the pathogenesis and transmission of infections.^7^ Health care workers (HCWs) carrying S. aureus in their nose or skin can play an important role in cross-contamination and thus MRSA related hospital acquired or sometimes community acquired infections.^3^ Screening and eradication of MRSA in hospital workers have been recommended as an important step in the prevention of MRSA infection. Moreover, proper knowledge about the prevalence and anti-microbial profile of this organism also help to decide proper empirical antibiotics in suspected patients infected with MRSA.^8^-^10^

Highest prevalence of MRSA in HCWs have been reported in Jordan (56%), Cyprus (55%) and Egypt (52%). While in some countries the prevalence of MRSA is low like 19% in Morocco and 12% in Lebanon. Akhtar N et al. found only 1.7% and Farzana K et al. found 14% prevalence of MRSA among HCWs in Pakistan.^11^-^13^ A study conducted by Moniri R et al. found 20% prevalence of MRSA among hospitalized patients in Pakistan.^14^ Determination of MRSA in nasal carriage of HCWs is very necessary, because they can play an important role in the transmission and cross-contamination of infections in hospitalized patients as well as the community. Because HCWs are also an interface between the community and hospital. As previous studies from different hospitals of Pakistan have found varying prevalence of MRSA in nasal flora of HCWs. We conducted this study to determine the prevalence of MRSA among HCWs of a tertiary care hospital of South Punjab Pakistan.

## METHODS

We included 225 HCWs including laboratory staff, doctors, nurses and paramedical staff using non-probability consecutive sampling in this descriptive cross-sectional study. The study was conducted in Nishtar medical university/Hospital Multan. By assuming prevalence of MRSA in HCWs of Pakistan 14% as reported by Farzana K et al.^13^, and taking α=5.0% this study sample size was 186 patients and we included 225 patients. The study setting was Nishtar Hospital Multan. The study was completed in 9 months from July-2016 to April-2017. HCWs having no signs of infections and any other systemic disease were included in this study. While HCWs taking anti-biotics presently and those who took any anti-biotic treatment in the last one month were excluded. Approval for this study from IRB of hospital was taken. Informed consent was taken from all HCWs by insuring them the confidentiality of their data.

We used sterile nasal swab sticks for sample collection. Mannitol sat agar was used in the laboratory for inoculation of swabs. All swabs were inoculated at 37^°^C temperature for 24 hours. Yellow colored colonies on mannitol agar that were coagulase positive and catalase positive were identified as S. aureus. For identification of MRSA we used Mueller- Hinton agar with Cefoxitin disc (30 μg) by Kirby-Bauer disc diffusion method. After incubation for 24 hours, diameter of colony size was measured for confirmation of MRSA, colony size ≤ 21 mm was labeled as MRSA and ≥22 mm was labeled as *methicillin sensitive S. aureus* (MSSA).

Kirby-Bauer diffusion disc technique as defined by CLSI guidelines was used for anti-biotic sensitivity testing. Antibiotics use were; oxacillin (1 µg), cefoxitin (30 µg), ceftriaxone (30 µg), amikacin (30 µg), co-amoxiclav (30 µg), azithromycin (15 µg), ciprofloxacin (30 µg), vancomycin (30 µg), and clindamycin (2 µg).

### Statistical analysis

Data analysis was done using statistical software SPSS v 23. Percentages were calculated for qualitative variables. Chi-square test was used for comparison of frequency of MRSA and MSSA between different KCWs by assuming p-Value ≤ 0.05 as significant difference.

## RESULTS

Baseline data of study patients regarding age, gender and working profession is depicted in Table-I. Mean age of patients was 32.55±12.45 years. There were 65.3% (147) female participants and only 34.7% (78) male participants. Regarding working profession, there were 61.3% (138) nurses, 21.3% (48) doctors, 13.8% (31) paramedics and 3.6% (8) laboratory technicians in this study.

**Table-I:** Baseline characteristics of participants.

Age	32.55±12.45
***Gender***	
Male	78 (34.7%)
Female	147 (65.3%)
***Working Profession***	
Doctors	48 (21.3%)
Nurses	138 (61.3%)
Paramedics	31 (13.8%)
Laboratory Technicians	8 (3.6%)

*S. aureus* was diagnosed in the nasal flora of 24% (54) participants, out of which 9.3% (21) were MRSA positive and remaining 14.7% (33) were MSSA positive. MRSA were diagnosed in 25% (2) lab. techs, in 10.86% (15) nurses, 6.2% (3) doctors and in only 3.2% (1) paramedics. MSSA was diagnosed in 2 (25%) Lab. Techs, in 15.94% (22) nurses, in 16.12% (4) paramedics and in 10.4% (5) doctors. This difference was not significant (p-value 0.79) Table-II.

**Table-II:** Prevalence of MRSA and MSSA among different HCWs.

Profession	MRSA	MSSA	Chi-square Statistics	p-Value
Doctors	3 (6.2%)	5 (10.4%)	1.007	0.79
Nurses	15 (10.86%)	22 (15.94%)		
Paramedics	1 (3.2%)	4 (16.12%)		
Laboratory Technicians	2 (25%)	2 (25%)		

Regarding antimicrobial sensitivity patterns for MRSA, amikacin and vancomycin were 100% (21) sensitive, clindamycin was 80.9% (17) sensitive and ciprofloxacin was 71.4% (15) sensitive. While oxacillin, Cefoxitin and ceftriaxone was almost 100% (21) resistant. Regarding MSSA, oxacillin, cefoxitin, amikacin, vancomycin and clindamycin were 100% (33) sensitive, ceftriaxone was sensitive in 75.7% (25) sensitive and ciprofloxacin was 84.8% (28) sensitive (Table-III).

**Table-III:** Antimicrobial sensitivity patterns.

Name of Antimicrobial	MRSA (N=21)		MSSA (N=33)	

	Sensitive	Resistant	Sensitive	Resistant
Oxacillin	0 (0.0%)	21 (100%)	33 (100%)	0 (0.0%)
Cefoxitin	0 (0.0%)	21 (100%)	33 (100%)	0 (0.0%)
Ceftriaxone	1 (4.8%)	20 (95.2%)	25 (75.7%)	8 (24.3%)
Amikacin	21 (100%)	0 (0.0%)	33 (100%)	0 (0.0%)
Co-amoxiclav	2 (9.5%)	19 (90.5%)	9 (27.3%)	24 (72.7%)
Azithromycin	6 (28.6%)	15 (71.4%)	10 (30.3%)	23 (69.7%)
Ciprofloxacin	15 (71.4%)	6 (28.6%)	28 (84.8%)	5 (15.2%)
Vancomycin	21 (100%)	0 (0.0%)	33 (100%)	0 (0.0%)
Clindamycin	17 (80.9%)	4 (19.1%)	33 (100%)	0 (0.0%)

## DISCUSSION

In our study, frequency of MRSA was 9.3%, while previous studies conducted in Pakistan have found 14.0% to 1.7% prevalence of MRSA among health care workers.^12^,^13^ Some other Asian countries have found a low prevalence of MRSA among HCWs. Khanal R et al. have reported 3.4% prevalence of MRSA in HCWs in western Nepal.^3^ Askarian M et al. reported 5.3% prevalence of MRSA HCWs working in Iran.^15^ Maliniet al. found 8% prevalence of MRSA in HCWs working in Banglore, India.^16^

In our study, overall prevalence of *S. aureus* was 24%. While Farzana et al. reported *S. aureus* prevalence in 48% of HCWs.^13^ Many studies have reported lower prevalence of S. aureus among HCWs, 12.4% prevalence in Libya, 17.5% in India, 20.8% in Palestine and 21% in Kuwait.^17^-^20^ While some other studies have reported higher prevalence such as 33.8% in Germany, 30% to 43.8% in USA and 31.0% in Iran.^15^, ^21^-^23^ The reason for these differences in the prevalence of MRSA and S. aureus among HCWs may be because all the studies were conducted in different regions of the world and prevalence may change due to differences in climate of these regions.

In our study, highest prevalence of MRSA was found among laboratory persons (25%) followed by nurses 10.86%. Khatri S et al. also found MRSA nasal carriage in highest proportion in lab technicians 10.5% followed by nurses 9.9% like our study.^24^ El-Aila NA et al. found highest prevalence of MRSA among nurses (30.4%), after that in doctors (16%).^25^ Shibabaw A et al. also found highest MRSA prevalence among nurses.^26^

In this study, amikacin and vancomycin were 100% sensitive for MRSA and MSSA. Clindamycin and ciprofloxacin was 80.9% and 71.4% sensitive for MRSA and 100% and 84.8% for MSSA respectively. While oxacillin and Cefoxitin were 100% resistant for MRSA and sensitive for MSSA. Khatri S et al. and El-Aila NA et al. also found vancomycin 100% sensitive for MRSA and MSSA and oxacillin 100% resistant for MRSA and 100% sensitive for MSSA.^24^,^25^

## CONCLUSION

Frequency of MRSA and MSSA is high among HCWs in Pakistan. Amikacin, vancomycin and clindamycin have high sensitivity for MRSA and can be used for empirical treatment of MRSA in suspected patients.

### Recommendations

Screening of health care workers of tertiary care medical institutions should be done to determine the frequency of MRSA and drug susceptibility for MRSA isolates in HCWs. This will help in early initiation of proper empirical antibiotics in MRSA infected patients.

### Author`s Contribution

**MKS**: Principal investigator, conceived, designed and wrote the manuscript.

**MSA & SI**: Did data collection, data compilation and did review.

**MARB:** Did review, and ready the final version of manuscript for publication.
